# Twelve-month safety, tolerability and susceptibility to adverse events of prophylactic migraine therapy with erenumab: a retrospective real-world study

**DOI:** 10.1186/s10194-022-01426-8

**Published:** 2022-05-11

**Authors:** Hannah Schenk, Dagny Holle, Michael Nsaka, Christoph Kleinschnitz, Martin Glas, Armin Scheffler

**Affiliations:** Department of Neurology and Center for Translational Neuro- and Behavioral Sciences (C-TNBS), West German Headache Center, University Hospital Essen, University Duisburg-Essen, Hufelandstr. 55, 45147 Essen, Germany

**Keywords:** CGRP antibody, Erenumab, Migraine, Safety and tolerability, Adverse event, Real-world

## Abstract

**Background:**

Erenumab is a monoclonal antibody (mAb) against the calcitonin gene related peptide (CGRP) receptor and is commonly used in migraine prophylaxis. Pivotal and open-label studies show a good safety and tolerability. However, little is known about possible predictors, dose dependence and time course of development of adverse events (AEs) during the treatment under real-world conditions.

**Methods:**

Clinical routine data of 128 patients with migraine treated in the West German Headache Center Essen were analyzed regarding AEs during a treatment interval of up to 12 months (3mo *n* = 128, 6mo *n* = 105, 9mo *n* = 74, 12mo *n* = 54). Patients obtained subcutaneous erenumab injections with either 70 mg or 140 mg per month. The occurrence and alterations of AEs were evaluated. All reported AEs, regardless of their severity, were included. AEs were graded using the common terminology criteria for adverse events (CTCAE). Possible parameters that could influence the occurrence of AEs (sex, episodic or chronic migraine, medication overuse headache, aura and the dosage of erenumab) were analyzed using the Chi-squared test, alpha adjustment was done using the Bonferroni’s correction (6 tests, adjusted alpha = 0.0083).

**Results:**

The proportion of patients who reported at least one AE were stable over the course of 12 months (after 3mo = 37%, 6mo = 36%, 9mo = 32%, 12mo = 35%). All reported AEs were grade 1 according to CTCAE with one exception (grade 2). Throughout the interval, five AEs were mostly reported: constipation, skin reactions, fatigue, sleep disturbances and nausea/emesis. Discontinuation of erenumab therapy was rarely caused by AEs (5/49). Increasing the dosage from 70 mg to 140 mg per month caused no higher frequency of AEs (Chi-squared test, *p* = 0.57). Significant more AEs were reported by females and by patients with aura (Chi-squared test, *p* < 0.001, respectively).

**Conclusion:**

In general, erenumab is well tolerated up to a treatment interval of 12 months and reported AEs rarely lead to discontinuation of therapy. A higher dosage does not increase the patient reported AEs. Furthermore, no habituation of AEs is observed. Nevertheless, females and patients with aura seem to be more prone to have AEs.

**Trial registration:**

No registration, retrospective analysis.

## Introduction

Monoclonal antibodies (mAB) against calcitonin gene related peptide (CGRP) or its receptor are the first specific preventive drugs for patients with episodic (EM) or chronic migraine (CM). Erenumab (Aimovig®, Novartis Europharm Limited, Switzerland) was licensed in July 2018 by the European Medicines Agency for adults with at least 4 migraine days per month [1]. The monoclonal fully humanized antibody targets the CGRP receptor [2]. Pivotal studies and open-label clinical trials (OLCT) have shown that beneficial effects seem to clearly outweigh adverse events (AE) regardless of the prescribed dosage of 70 mg or 140 mg per month up to 5 years [3–5]. Clinical trials regarding efficacy and tolerability of erenumab therapy involved patients with migraine who had formerly discontinued up to 4 preventive medications [4]. Therefore, drug-resistant patients with more than 4 failed preventatives were not observed. However, in routine clinical settings, these is the predominant treatment population. Sufficient data about AEs in this cohort regarding development and predictors for AEs are rare.

The aim of this study was to provide real-world data about drug resistant migraine patients under therapy with erenumab regarding AEs with focus on the possible habituation effect of AEs, the occurrence of AEs in relation to the respective erenumab dosage, possible predictors of AEs and long-term effects up to 1 year.

## Methods

Clinical routine data of 128 patients with EM or CM who obtained treatment with erenumab for up to 12 months at the West German Headache Center, Department of Neurology, University Hospital Essen, in Germany between November 2018 and November 2020 was analyzed. The analysis was permitted by the independent ethics committee of the University Hospital Essen (19–9004-BO). Patients were included in the analysis by meeting these subsequent criteria: a) the diagnosis of migraine according to the International Classification of Headache Disorders, 3rd edition [6], b) completion of at least 3 months of treatment with erenumab with a documentation to the respective timepoint if AEs occurred or not, c) due to reasons of reimbursement by the German statutory health insurance, all treated patients had tried at least four (in case of EM) or five (in case of CM) approved prophylactic drugs in the past without sufficient treatment effects, had discontinued those due to AEs or were not eligible for intake due to contraindication. Listed mandatory drug classes are the following: anticonvulsants (topiramate), betablockers (metoprolol, propranolol), calcium channel blockers (flunarizine), tricyclic antidepressants (amitriptyline) and onabotulinumtoxin A (only in case of CM).

AEs reported by patients were evaluated after three, six, nine and twelve months of treatment. All AEs were rated using the U.S. National Cancer Institute’s common terminology criteria for adverse events (CTCAE v5.0) [7] and compiled. The AE ‘constipation’ included all patients with emerging difficulties emptying their bowls or reporting hardened feces. Following symptoms were summarized as ‘skin reaction’: swelling, rashes, wound healing disturbances or pruritus. The indication of increased tiredness was categorized as ‘fatigue’ and difficulties falling asleep and staying asleep as ‘sleep disturbances’.

Most patients were prescribed 70 mg per month at the beginning of the therapy interval. Every 3 months a reassessment was conducted regarding efficacy, AEs and changes of migraine characteristics. Whenever the therapy efficacy was evaluated as insufficient, the dosage was increased to 140 mg or the therapy was changed. The dosage was increased when following criteria were fulfilled: a) efficacy insufficient (lack of response regarding monthly headache and/or migraine days) b) good tolerability allowed the increase c) patients satisfaction was improvable.

The criteria for discontinuation of therapy was the lack of response regarding monthly headache and/or migraine days, the occurrence of AEs and/or patient’s dissatisfaction.

Furthermore, cumulative timepoints with and without AEs over the therapy interval up to 12 months were merged. Thereby, parameters which could possibly predict an AE (sex, migraine type (EM/CM), medication overuse headache (MOH), aura and dosage per month (70 mg or 140 mg) were analysed. Differences in the occurrence of AEs regarding the respective parameter were analysed using the Chi-squared test. Alpha adjustment was performed using the Bonferroni’s correction for multiple tests (six tests, adjusted alpha = 0.0083). The data was analysed with SPSS software (IBM SPSS Statistics for Windows, Version 27.0. Armonk, NY, USA) and Excel (Microsoft Corporation, Version 1809, Redmond, Washington, USA).

## Results

One hundred twenty-eight patients with migraine (3mo *n* = 128, 6mo *n* = 105, 9mo *n* = 74, 12mo *n* = 54) were analysed. At the first timepoint, females dominated the cohort with 83% (106 female, 22 male), the mean age was 49.2 years (min: 21 years max: 94 years). A dosage increase from 70 mg to 140 mg was conducted for 40 patients after 3 months (31%), 19 patients after 6 months (18%) and 8 patients after 9 months (11%). Each patient who reported AEs stated an average of 1.6 (SD: 1.22) different AEs. Without considering the dosage (70 mg or 140 mg per month), 37% (*n* = 47) of all patients reported AEs after 3 months, 36% (*n* = 38) after 6 months, 32% (*n* = 24) after 9 months and 35% (*n* = 19) after 12 months. Seventy-seven patients had other comorbidities, most common were hypothyroidism (15%, *n* = 19), bronchial asthma (10%, *n* = 13), depression (9%, *n* = 12) and arterial hypertension (5%, *n* = 6).

**Fig. 1:**
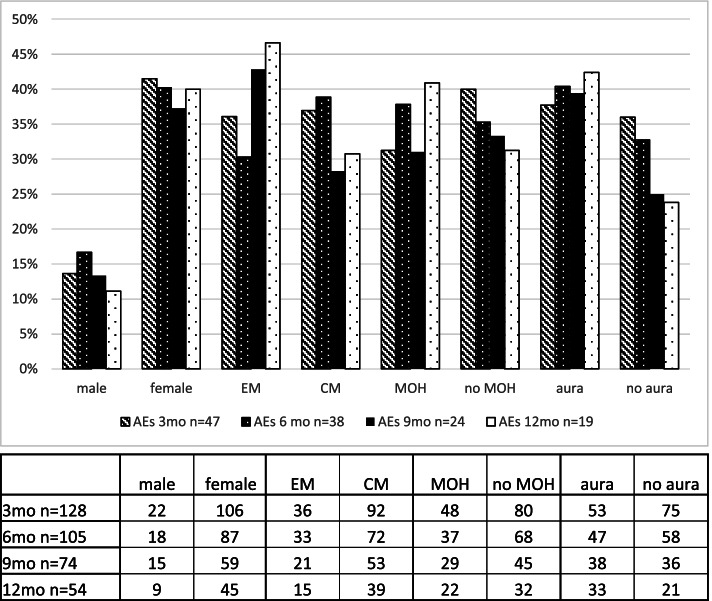
Adverse events depending on patient characteristics. Patients’ characteristics like sex, medication-overuse headache and aura. Proportion of patients with the respective characteristics who reported AEs

Over the 12-month treatment interval, data was analysed with the intention to identify possible parameters that could influence the frequency of AEs. Males (3mo = 14%, *n* = 3 of 22; 6mo = 17%, *n* = 3 of 18; 9mo = 13%, *n* = 2 of 15; 12mo = 11%, *n* = 1 of 9) stated significantly less AEs than females (3mo = 42%, *n* = 44 of 106; 6mo = 40%, *n* = 35 of 87; 9mo = 37%, *n* = 22 of 59; 12mo = 40%, *n* = 18 of 45) throughout the entire observation period (Chi-squared test: *p* < 0.001, Fig. 1). Furthermore, patients who suffered under an aura reported a significant higher frequency of AEs than patients without an aura (Chi-squared test: *p* < 0.001) over the 12-month period. For other parameters, no significant difference was observed (EM/CM: *p* = 0.67; MOH/noMOH: *p* = 0.78).

**Fig. 2:**
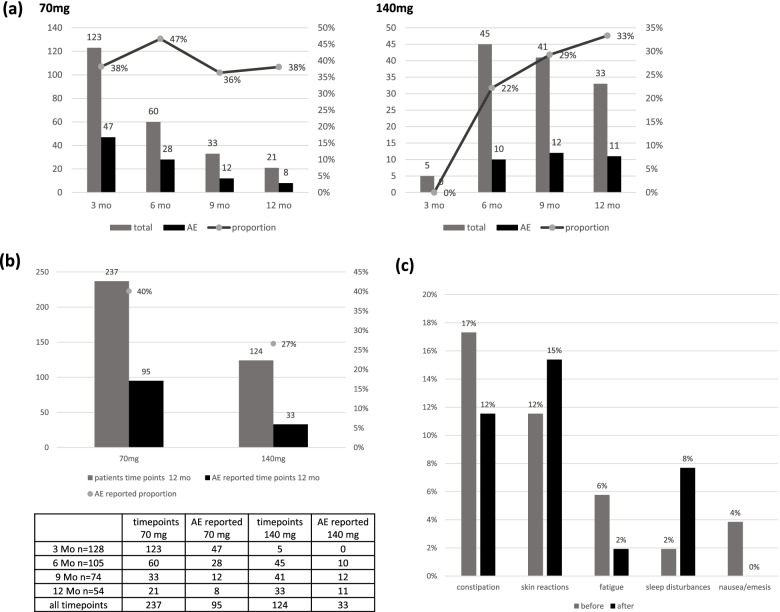
Cumulative dose-related adverse events. Reported AEs with 70 mg or 140 mg erenumab per month **(a),** cumulative timepoints and timepoints with reported AEs depending on the respective dosage over the therapy up to 12 months **(b).** Reported AEs before and after increasing erenumab dosage to 140 mg per month **(c)**

The total of reported AEs and correlations between the different monthly dosage (70 mg or 140 mg) were evaluated (Fig. 2a). Almost all patients started the erenumab therapy with a 70 mg monthly dosage (70 mg *n* = 123 (96%); 140 mg *n* = 5 (4%)) due to internal hospital requirements. In relation to all occurred AEs over the 12 months, patients treated with 70 mg erenumab per month reported more AEs (40%, *n* = 95 of 237) than patients treated with 140 mg erenumab per month (27%, *n* = 33 of 124; Fig. 2b). Nevertheless, no significant difference in the frequency of reported AEs was observed to the adjusted alpha = 0.0083 (Chi-squared test: *p* = 0.011).

**Fig. 3:**
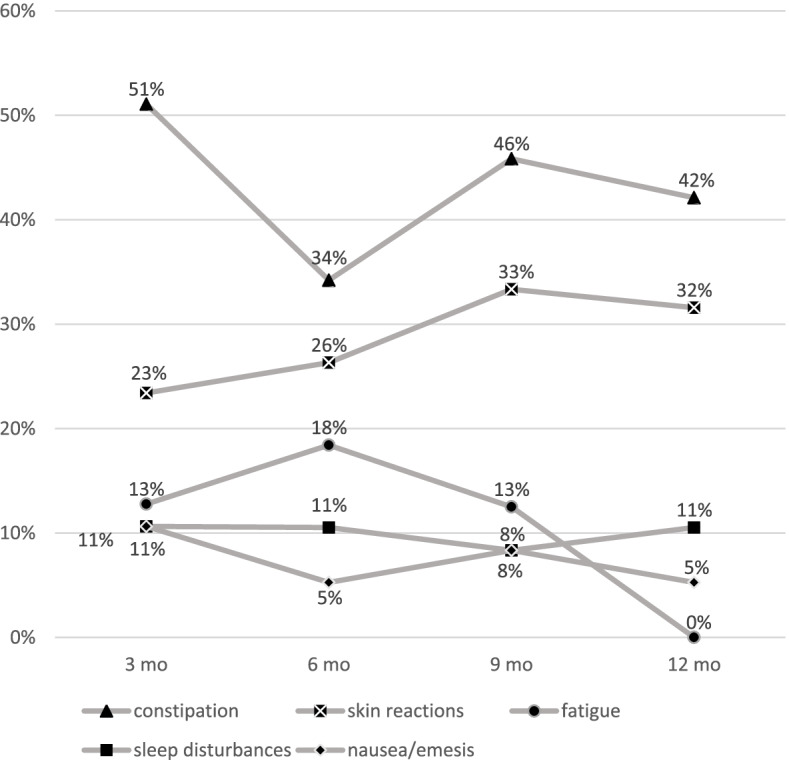
Specific adverse events. Specific adverse events in proportion to all reported AEs over 12 months

The five prevalent specific AEs in this cohort were constipation, skin reactions, fatigue, sleep disturbances and nausea/emesis (Fig. 3). The data showed that after 3 months of treatment with erenumab, constipation occurred in 51% (*n* = 24) of cases. Even after 12 months, this side effect remained the most prevalent at 42% (*n* = 8). Overall, each specific AE proceeded predominantly stable over the duration of the therapy, except for skin reactions, demonstrating a slight increase and in contrast nausea/emesis a slight reduction of occurred events (Fig. 3).

All patients that underwent a dosage increase from 70 mg to 140 mg throughout the interval (*n* = 67) were pooled. Twenty-two patients (33%) before and 19 patients (28%) after the dosage increase described AEs. No significant difference was observed (Chi-squared test: *p* = 0.57). Regarding specific AEs, constipation, fatigue and nausea/emesis showed no exacerbation trend and slightly decreased under the higher dose (Fig. 2c). In detail (*n *= specific AE, % of all reported AEs): constipation (before *n* = 9 (17%), after *n* = 6 (12%)), fatigue (before *n* = 3 (6%), after *n* = 1 (2%)), nausea/emesis (before *n* = 2 (4%), after *n* = 0 (0%)). More skin reactions were observed by patients after altering their dosage (from 6 patients (12%) using 70 mg to 8 patients (15%) using 140 mg monthly). Similar tendencies were seen in the case of sleep disturbances, involving one patient receiving 70 mg and four patients receiving 140 mg. Most of these patients reported the respective AEs for the first time after increasing the dosage to 140 mg, skin reactions with 140 mg appeared for the first time in four out of eight patients and sleep disturbances in three out of four.

Reasons for the withdrawal from the treatment were analysed. The lack of efficacy caused at least 84% (*n* = 41 of 49) of all dropouts (3mo *n* = 16 of 19 (84%), 6mo *n* = 16 of 19 (84%), 9mo *n* = 9 of 11 (82%)) and is therefore the main reason of discontinuation. Regarding AEs, one patient interrupted the therapy due to gastrointestinal problems and two patients because of insufficient efficacy and other AEs such as nausea and dyspnea after 3 months of treatment. At the end of 6 months, three patients discontinued because of hair loss, obstipation, pruritus, heart palpitations and skin rash. After 9 months, one patient discontinued due to severe wound healing disturbances, which was already described elsewhere [8], as well as one patients’ therapy has been discontinued due to insufficient efficacy along with AEs including fatigue and nausea/emesis.

All except one reported AEs were mild and were grade 1 according to the CTCAE. Only the wound healing disorder described above reached grade 2. Three of five patients who had discontinued therapy solely because of AEs reported no more complaints afterwards (constipation, pruritus, wound healing disturbances). One patient had received a different CGRP antibody afterwards. Under the new therapy the AEs were still present (unspecific gastrointestinal problems).

Since AEs could also be symptoms of other comorbidities, patients with depression, bronchial asthma and those treated for postmenopausal symptoms were reviewed. None of the 12 patients with depression reported sleep disturbances, only one of the asthma patients reported skin reactions. These patients did not show an unusually high number of AEs that could be attributed to the comorbidity. One patient was treated for postmenopausal symptoms. She reported constipation, flushing and orthostatic problems, which could be symptoms of the underlying disease.

## Discussion

CGRP mAB therapy is becoming increasingly popular and offers good tolerability. But there is still a lack of sufficient data on tolerability under real-world conditions. Possible predictors of AEs, especially in the group of drug-resistant patients with migraine are unknown. In Germany, CGRP mAB are primarily used in these severely affected patients. Thus, this study focused on development of AEs, on possible habituation effects over time, on predictors for AEs and on association between the different dosage of erenumab (70 mg or 140 mg per month) under real-world conditions.

The occurrence of the AEs was slightly less than in randomized controlled clinical trials (EM 140 mg = 55%, CM 70 mg = 44% and 140 mg = 47%) [4, 5]. In our study 37% of migraine patients reported AEs after 3 months. These rates of AEs remained stable with 36% after 6 months, 32% after 9 months and 35% after 12 months, indicating no clear signs of a habituation effect over a period of 12 months.

The low rates of discontinuation due to AEs in this study support the good long-term tolerability. Although the overall rate of withdrawal from the treatment is higher compared to pivotal studies (e.g., in the LIBERTY study 2%) [4, 5] the rates are steady throughout, with 19 patients discontinuing after 3 months (15%), 19 patients after 6 months (18%) and 11 patients after 9 months (15%). We did not analyse the proportion of withdrawals after 12 months, because of an uncertainty of the data due to a mandatory treatment break after 12 months, recommended by European [1] and German [9] guidelines. During the analysed timepoints, the treatment of most patients was interrupted because of lacking efficacy and not as a consequence of AEs. In detail, one patient withdrew after 3 months merely due to AEs, three patients after 6 months and one patient after 9 months. One of those patients presented severe wound healing disturbances after 9 months of erenumab therapy, which was already described elsewhere [7].

A higher number of AEs emerged in patients using 70 mg per month compared to the higher dosage of 140 mg per month. When all timepoints of the 12-month observation period are merged, 40% stated AEs when receiving 70 mg and just 27% receiving 140 mg erenumab per month (Fig. 2b), but the slight significant difference (Chi-squared test: *p* = 0.011) was not significant after Bonferroni’s correction (adjusted alpha = 0.0083). Thus, no dosage dependence was observed. However, the effect could be influenced by study requirements for a dosage increase, which was conducted whenever the patients’ reevaluation showed improvable efficacy and rather no or no significant AEs occurred. Furthermore, no dosage dependence could be detected when individual AEs are taken into consideration. No distinct association between the higher dosage and a higher incidence was seen (Chi-squared test: *p* = 0.57, Fig. 2c). Further clinical studies will be needed to prove, whether these observations are purely coincidental because of the small sample size.

During the observation period, a fluctuating dispersion of reported AEs was seen in the different parameters (MOH, EM/CM, aura) depending on the time points (Fig. 1). A part of these fluctuations could also be a result of the discontinuation of patients with the respective parameter at the respective timepoint. Reasons for the missing follow-up were discontinuation of treatment because of the limited therapy efficacy/AEs or an incomplete treatment interval at the time of evaluation. Nevertheless, taking the whole observation period into consideration, patients with aura showed a significant higher frequency of AEs than patients without an aura (Chi-squared test: *p* < 0.001), indicating a possible risk factor for AEs. However, there is no conclusive explanation for increased AEs in patients with aura in the studies to date. In addition, our data also showed a consistently higher frequency of AEs reported by females (Chi-squared test *p* < 0.001). Other studies also suggest a gender-specific efficacy with the male sex (with CM), suggesting a positive predictor for responsiveness to erenumab [10]. This could be explained by the lower prevalence of migraine among males [11], resulting in a smaller sample size. In contrast, it could imply a gender-specific association with AEs and therefore a higher susceptibility to AEs in females during an erenumab therapy. Supporting gender-specific differences animal studies showed a lower density of CGRP receptors in the trigeminal ganglion and medulla of female rats and also a modification of the CGRP effect by ovarian hormones (especially estradiol). This could indicate an altered CGRP pathway of the trigeminal system in females (reviewed in [12]). Further research including possible therapy adaptations for the female sex and for patients with aura may be considered.

In the pivotal clinical trials and OLCT, the leading AEs were upper respiratory tract infections, nasopharyngitis, injection side pain and constipation [3–5]. In our cohort, constipation (3mo *n* = 24, 6mo *n* = 13, 9mo *n* = 11, 12mo *n* = 8) was the most documented AE, followed by skin reactions (3mo *n* = 11, 6mo *n* = 10, 9mo *n* = 8, 12mo *n* = 6) (Fig. 3). In another observational study, the significantly higher number of constipation events was explained with patients’ expectations because of the explanatory talk by the attending doctor [13].

Nevertheless, there are some indications that erenumab could cause specific AEs. It was shown that CGRP influences intestinal motility as well as gastric acid secretion [14]. Further, animal studies suggested a dominant role of CGRP in intestinal motility [15] and vasodilatation [16]. In humans, co-localization of the two components of the CGRP receptor (calcitonin receptor-like receptor (CLR); receptor activity-modifying protein 1 (RAMP1)) was observed in the enteric nerve plexus of the stomach, ileum and colon [17]. The specific mechanisms of CGRP in the human gastrointestinal tract and its function require further research. Nonetheless, the inhibition of this system via CGRP antibodies might interfere with the physiological cycle of digestion leading to constipation. Additionally, an ileus under erenumab therapy after surgery has been described which could confirm the association between obstipation and the CGRP receptor blockade by erenumab [18].

A regulatory function of CGRP in the skin is known [19], and a case of severe wound healing disorder has also been described. It is suggested that impaired healing is caused by the inhibition of CGRP and consequently the absent downregulating effects of CGRP on the endothelial proinflammatory cytokines [8, 20]. However, an accumulation of wound healing disorders has not been reported so far. Nevertheless, due to the role of CGRP in skin, mild skin reaction could be caused by CGRP receptor blockade. With regard to fatigue and sleep disturbances, there is no evidence so far that these symptoms could be specific AEs of erenumab.

Additionally, AEs could not be clearly separated from symptoms of the underlying comorbidity in every case. This may lead to a possible biased evaluation. Nevertheless, patients with depression and bronchial asthma reported no increased AEs which could be clearly associated with their comorbidities, indicating only a small proportion of reported AEs are associated with other comorbidities. However, not all AEs could be explained due to the erenumab effect.

A limitation of this single-center study, besides its retrospective nature without a placebo group, is the predominantly subjective acquired data, based on patients’ questionnaires and statements. Nevertheless, real-world adherence is necessary to confirm long-term tolerability especially in a clinical routine setting and in patients with drug resistant migraine.

## Conclusion

Our data suggests long-term safety and tolerability during a treatment interval of up to 12 months in the cohort of patients with drug-resistant migraine. Despite the high absolute and relative number of reported AEs, AEs hardly lead to discontinuation of therapy and show no dose dependency. The data also suggests that females and patients with aura are associated with a worse AE profile. Although these findings still need to be verified in a randomised controlled trial, a gender and migraine-specific therapy regime could be necessary.

## Data Availability

The datasets used and/or analysed during the current study are available from the corresponding author on reasonable request.

## Abbreviations

AE: Adverse event
CGRP: Calcitonin gene-related peptide
CM: Chronic migraine
CTCAE: Common terminology criteria for adverse events
EM: Episodic migraine
mAB: Monoclonal antibody
MOH: Medication-overuse headache
OLCT: Open-label clinical trial
SD: Standard deviation

## References

[1] Sacco S, Bendtsen L, Ashina M (2019). European headache federation guideline on the use of monoclonal antibodies acting on the calcitonin gene related peptide or its receptor for migraine prevention. J Headache Pain.

[2] Shi L, Lehto SG, Zhu DXD (2016). Pharmacologic characterization of AMG 334, a potent and selective human monoclonal antibody against the calcitonin gene-related peptide receptor. J Pharmacol Exp Ther.

[3] Ashina M, Goadsby PJ, Reuter U (2019). Long-term safety and tolerability of erenumab: three-plus year results from a five-year open-label extension study in episodic migraine. Cephalalgia.

[4] Reuter U, Goadsby PJ, Lanteri-Minet M (2018). Efficacy and tolerability of erenumab in patients with episodic migraine in whom two-to-four previous preventive treatments were unsuccessful: a randomised, double-blind, placebo-controlled, phase 3b study. Lancet.

[5] Tepper S, Ashina M, Reuter U (2017). Safety and efficacy of erenumab for preventive treatment of chronic migraine: a randomised, double-blind, placebo-controlled phase 2 trial. Lancet Neurol.

[6] Headache Classification Committee of the International Headache Society (2013). The international classification of headache disorders, 3rd edition (beta version). Cephalalgia.

[7] National Cancer Institute Common Terminology Criteria for Adverse Events (CTCAE), US Department of Health and Human Services, National Institutes of Health, National Cancer Institute. https://ctep.cancer.gov/protocoldevelopment/electronic_applications/ctc.htm.

[8] Wurthmann S, Nägel S, Hadaschik E (2020). Impaired wound healing in a migraine patient as a possible side effect of calcitonin gene-related peptide receptor antibody treatment: a case report. Cephalalgia.

[9] Diener H-C, Förderreuther S, Gaul C (2020). Prophylaxe der Migräne mit monoklonalen Antikörpern gegen CGRP oder den CGRP-Rezeptor, Ergänzung der S1-Leitlinie Therapie der Migräneattacke und Prophylaxe der Migräne. DGNeurologie.

[10] Barbanti P, Aurilia C, Cevoli S (2021). Long-term (48 weeks) effectiveness, safety, and tolerability of erenumab in the prevention of high-frequency episodic and chronic migraine in a real world: results of the EARLY 2 study. Headache.

[11] Victor TW, Hu X, Campbell JC (2010). Migraine prevalence by age and sex in the United States: a life-span study. Cephalalgia.

[12] Labastida-Ramírez A, Rubio-Beltrán E, Villalón CM (2019). Gender aspects of CGRP in migraine. Cephalalgia.

[13] Ornello R, Casalena A, Frattale I (2020). Real-life data on the efficacy and safety of erenumab in the Abruzzo region, Central Italy. J Headache Pain.

[14] Rekik M, Delvaux M, Frexinos J, Bueno L (1997). The Calcitonin Gene-Related Peptide Activates Both cAMP and NO Pathways to Induce Relaxation of Circular Smooth Muscle Cells of Guinea-Pig Ileum. Peptides.

[15] Clifton MS, Hoy JJ, Chang J (2007). Role of calcitonin receptor-like receptor in colonic motility and inflammation. Am J Physiol Gastrointest Liver Physiol.

[16] Kono T, Koseki T, Chiba S (2008). Colonic vascular conductance increased by Daikenchuto via calcitonin gene-related peptide and receptor-activity modifying protein 1. J Surg Res.

[17] Cottrell GS, Alemi F, Kirkland JG (2012). Localization of calcitonin receptor-like receptor (CLR) and receptor activity-modifying protein 1 (RAMP1) in human gastrointestinal tract. Peptides.

[18] Frattale I, Ornello R, Pistoia F (2021). Paralytic ileus after planned abdominal surgery in a patient on treatment with erenumab. Intern Emerg Med.

[19] Kim YJ, Granstein RD (2021). Roles of calcitonin gene-related peptide in the skin, and other physiological and pathophysiological functions. Brain Behav Immun Health.

[20] Hou Q, Barr T, Gee L (2011). Keratinocyte expression of calcitonin gene-related peptide β: implications for neuropathic and inflammatory pain mechanisms. Pain.

